# Association between adipocytokines and diabetic retinopathy: a systematic review and meta-analysis

**DOI:** 10.3389/fendo.2023.1271027

**Published:** 2023-10-06

**Authors:** Yanhua Jiang, Huaying Fan, Jing Xie, Yao Xu, Xin Sun

**Affiliations:** ^1^Department of Ophthalmology, Fourth People’s Hospital of Shenyang, Shenyang, China; ^2^Department of Endocrinology and Metabolism, First Affiliated Hospital of Soochow University, Suzhou, China; ^3^Department of Ophthalmology, First Affiliated Hospital of Soochow University, Suzhou, China

**Keywords:** adipocytokines, diabetic retinopathy, DR, leptin, chemerin

## Abstract

**Background:**

Diabetic retinopathy (DR) is a common complication of diabetes. The adipocytokines are closely associated with the occurrence and development of diabetes and its related complications. Literature confirms that the level of adiponectin in patients with DR is significantly higher; however, the relationship between other adipocytokines (leptin, chemerin, apelin, and omentin-1) and DR remains unclear.

**Aim:**

This study aimed to systematically evaluate the association between adipocytokines (leptin, chemerin, apelin, and omentin-1) and DR.

**Methods:**

The PubMed, Web of Science, Embase, EBSCO and Willy databases were used to search for potential studies with keywords such as “diabetic retinopathy” or “DR” in combination with the terms “leptin,” “chemerin”, “apelin” or “omentin-1” in the search titles or abstracts. Standardized mean differences (SMD) with corresponding 95% confidence intervals (CIs) were determined as the results of the meta-analysis.

**Results:**

After screening, 18 articles were included in the meta-analysis including 750 DR cases and 993 controls. Leptin and chemerin levels in patients with DR were significantly higher than those in the control group (SMD: 0.68, 95% CI [0.1, 1.26]; SMD: 0.79, 95% CI [0.35, 1.23]). The omentin-1 levels in patients with DR were significantly lower than those in the controls (SMD: –0.85, 95% CI [–1.08, –0.62]).

**Conclusions:**

To the best of our knowledge, this is the first meta-analysis to evaluate the leptin, chemerin, apelin, and omentin-1 levels in patients with DR. Further high-quality studies are warranted to support the association between these adipocytokines and DR.

**Systematic review registration:**

https://www.crd.york.ac.uk/PROSPERO/display_record.php?RecordID=443770, identifier CRD42023443770.

## Introduction

Diabetic retinopathy (DR) is a common complication of diabetes. A recently published meta-analysis involving 59 studies revealed that the global prevalence of DR in individuals with diabetes was 22.27% (1). By 2020, the estimated number of adults affected by DR worldwide was approximately 103.1 million, which is projected to increase to 160.5 million by 2045. Approximately 16 million individuals in the United States will reportedly experience DR by 2050 (2). In Europe, it is estimated that patients with DR will increase from 6.4 million in 2019 to 8.6 million by 2050, with 30% of the population requiring close monitoring or treatment (3). DR is clinically divided into non proliferative DR (NPDR) and proliferative DR (PDR) based on the presence or absence of retinal neovascularization. According to these findings, DR can be attributed to the detrimental effects of hyperglycemia on the retinal capillaries, resulting in edema, bleeding, and obstruction of the surrounding tissues (4). Concurrently, the elevated blood glucose levels induce retinal ischemia, hypoxia, and generation of neovascular proliferators, ultimately resulting in the onset of retinopathy. Furthermore, an extended duration of diabetes is positively correlated with an increased incidence of DR. The increase in the number of risk factors at target correlates with better cardiovascular-free survival in patients with type 2 diabetes at high cardiovascular risk (5). Thus, early intervention in the management of diabetes is of paramount significance (6).

**Figure 1:**
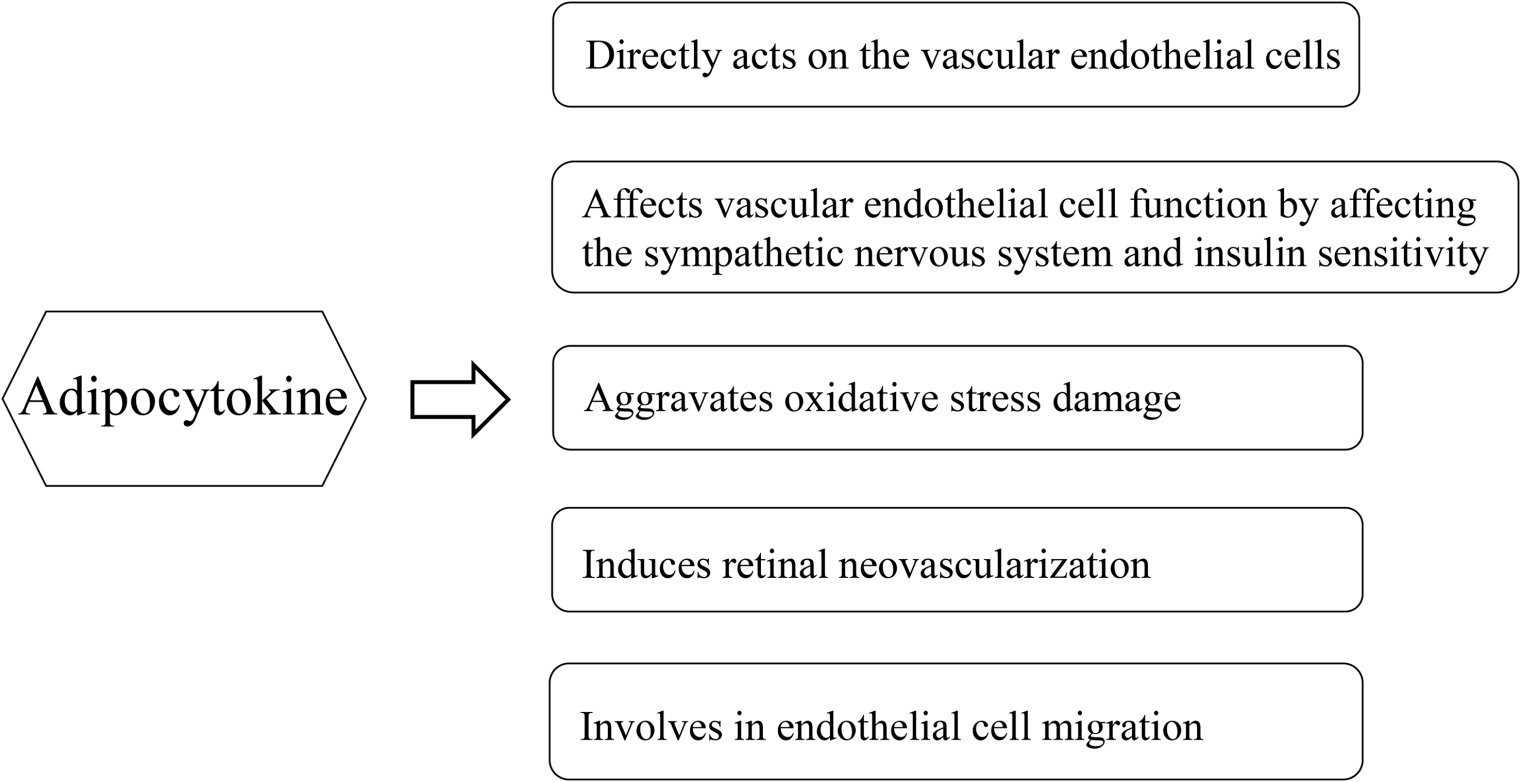
Multifactorial effects of adipocytokines in the pathology of diabetic retinopathy.

Adipose tissue has traditionally been considered a long-term energy storage organ, and researchers are now realizing its crucial role in metabolism. Adipose tissue is generally believed to be an endocrine organ that secretes various bioactive substances, and the factors secreted by the adipose tissue are collectively referred to as adipocytokines, which include adiponectin, leptin, apelin, chemerin, and omentin-1. These adipocytokines are closely associated with the occurrence and development of DR (**Figure 1**). Adipose factors can directly act on the vascular endothelial cells through the blood circulation. It can also indirectly affect vascular endothelial cell function by affecting the sympathetic nervous system and insulin sensitivity (7). Adipocytokines are mediators linking adipose tissue and inflammation, which in turn generate an unbalance in oxidative stress levels. In the development of diabetic retinopathy, as the retinal tissue is highly susceptible to damage mediated by oxidative stress due to its high concentration of polyunsaturated fats (8). Although literature has confirmed that the level of adiponectin in patients with DR is significantly high (9), the relationship between other adipocytokines and DR remains unclear. Leptin, which is a peptide-active factor specifically secreted by the human adipose tissue, plays important regulatory roles in human growth and development, immune function, cellular inflammatory responses, angiogenesis, platelet aggregation, epithelial cell proliferation, and migration (10). Some studies demonstrated that the leptin levels in the serum and aqueous humor of patients with DR were significantly increased compared to those of the control group (11, 12). However, Malik et al. demonstrated that the serum leptin levels of patients with DR were lower than those of the control group (13). Moreover, the relationship between chemerin, apelin, and omentin-1, and DR is controversial. Therefore, this study aimed to systematically evaluate the association between adipocytokines (leptin, chemerin, apelin, and omentin-1) and DR.

## Methods

### Literature search

Our meta-analysis was registered in PROSPERO under the accession number CRD42023443770. PubMed, Web of Science, Embase, EBSCO and Willy databases were used to search for potential studies. The following search terms were used for the title or abstract: “diabetic retinopathy” and “DR” in combination with the terms: “leptin,” “apelin,” “chemerin,” or “omentin-1”. The search period was from 1980 to 2023. We simultaneously traced the references of the collected relevant literature, searched for studies that met the inclusion criteria, and eliminated duplicate studies. This meta-analysis was performed in accordance with the Preferred Reporting Items for Systematic Reviews and Meta-Analyses (PRISMA) guidelines; the PRISMA list is provided in the **Supplementary File (S1)** (14).

### Inclusion criteria

Only studies that met the following criteria were included in this meta-analysis: (1) studies with patients with DR, (2) studies reporting the correlation between the levels of adipocytokines (leptin, apelin, chemerin, and omentin-1) and DR, (3) studies with > 20 samples, and (4) studies written in English.

### Exclusion criteria

(1) Reviews, case reports, comments, and animal experimental studies; (2) duplicate or repeat publications; and (3) literature with incomplete data.

### Data extraction and risk of bias

Based on the inclusion and exclusion criteria, two researchers independently read the titles, abstracts, and full texts. In cases of disagreement, a third researcher was asked to intervene and make the final decision. Data were extracted, including the author, year, survey area, sample size, and indicators.

The Newcastle-Ottawa Scale (NOS) is a risk of a bias assessment tool for observational studies recommended by the Cochrane Collaboration (15). The quality of the included studies was evaluated according to the NOS. The NOS includes three aspects: the selection method of the case and control groups, comparability of the case and control groups, and evaluation method of exposure. The NOS ranged from zero to nine stars, and quality was based on star scores.

### Statistical analysis

The results of the systematic analysis are presented as standardized mean differences (SMDs) with corresponding 95% confidence intervals (CIs). The meta-analysis was conducted using Stata 12.0 software (College Station, TX, USA). First, a heterogeneity test was performed using I^2^. If I^2^ < 50%, a fixed effects model is used; If I^2^ ≥ 50%, heterogeneity is significant and a random effects model is used. The stability of the meta-analysis results was evaluated using a sensitivity analysis. Low-quality literature and the impact of a single study on the overall research results were excluded from each study. Publication bias was tested using Begg’s and Egger’s tests. A *p* value< 0.05 was considered statistically significant.

## Results

**Figure 2:**
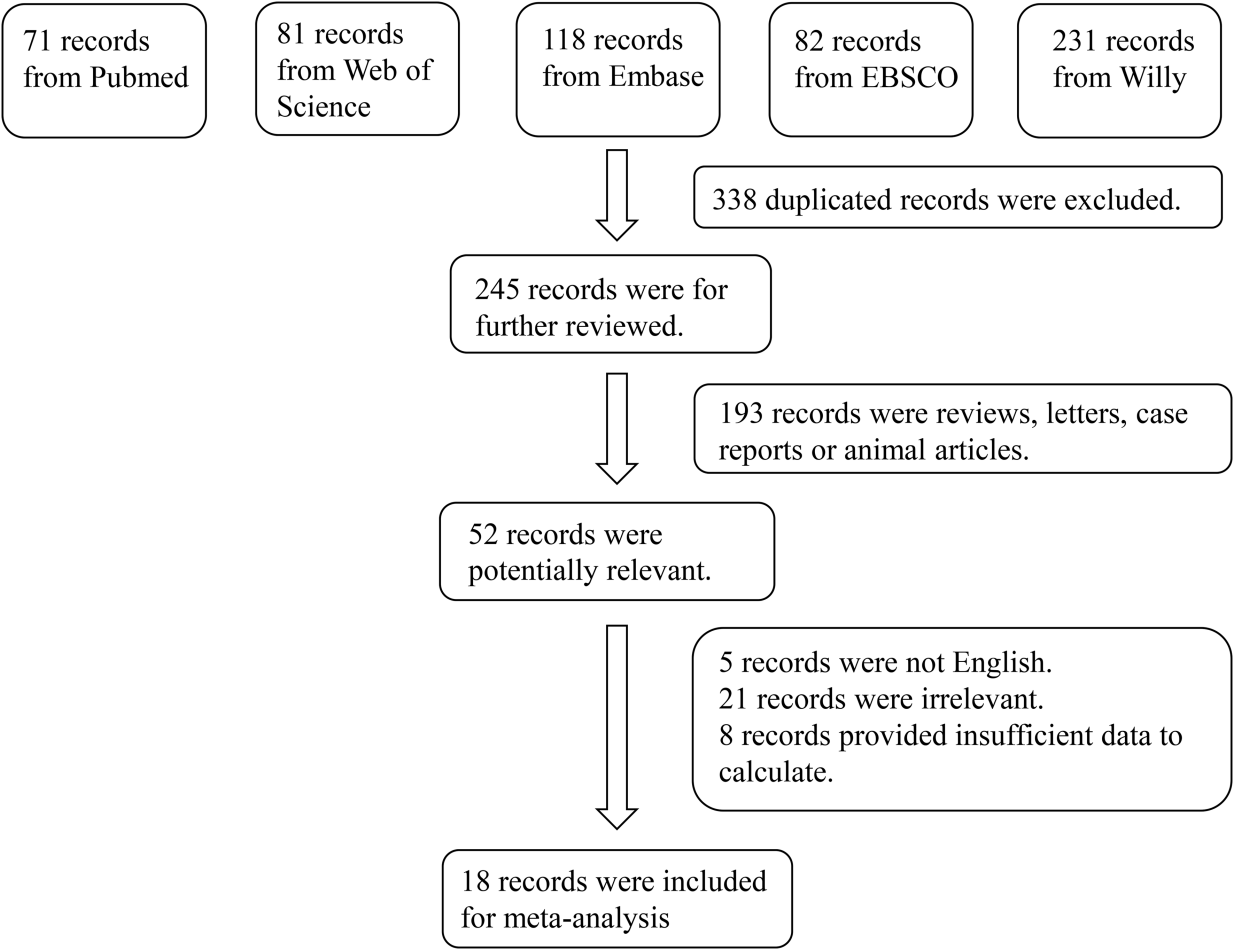
Flowchart of the detailed procedure for the inclusion or exclusion of selected studies.

In total, 583 studies were retrieved from the PubMed, Web of Science, Embase, EBSCO and Willy. After screening, 18 articles were included in the meta-analysis (11–13, 16–30). The reasons for inclusion during the full-text selection are shown in **Figure 2**. Altogether, 18 articles included 750 DR cases and 993 controls. The characteristics of the selected studies are summarized in **Table 1**. Overall, in accordance with the suggested criteria for the Selection, Comparability, and Exposure categories of the Newcastle-Ottawa Scale, the studies included in this meta-analysis were of acceptable quality.

**Table 1:** Study characteristics of the published studies included in the meta-analysis.

Author	Publication Year	Study Period	Region	Study design	Detection method	Case(n)	Control(n)	Case	Control	Indicator
Gariano RF	2000	–	USA	Case-control study	Radioimmunoassay	39	32	25.2 ± 6.9	12.1 ± 1.3	Leptin
Uckaya G	2000	–	Turkey	Case-control study	Radioimmunoassay	37	33	13.6 ± 17.03	5.8 ± 3.7	Leptin
Hernández C	2004	–	Spain	Case-control study	ELISA	25	32	23 ± 15.4	20.1 ± 18.8	Leptin
Yonem A	2009	February 2007 – September 2007	Turkey	Case-control study	ELISA	38	41	4.22 ± 2.84	4.45 ± 3.68	Apelin
Tao Y	2010	–	China	Prospective study	ELISA	55	34	16.9 ± 12.5	18.1 ± 13.4	Apelin
Tasci E	2011	–	Turkey	Case-control study	ELISA	9	51	27 ± 30	7.47 ± 9.05	Leptin
Dossarps D	2013	February 2008 – November 2010	France	Case-control study	ELISA	69	110	22.84 ± 18.75	19 ± 27	Leptin
Du J	2014	May 2012 – June 2013	China	Case-control study	ELISA	39	30	4.04 ± 1.24	3.7 ± 1.49	Apelin
Wan W	2015	–	China	Case-control study	ELISA	144	60	149.19 ± 39.03	184 ± 47	Omentin-1
Du J	2015	May 2012 – April 2013	China	Case-control study	ELISA	35	25	136.57 ± 27.88	113 ± 20	Chemerin
Li J	2016	–	China	Case-control study	ELISA	38	25	4.93 ± 4.59	2.53 ± 3.06	Chemerin
Malik TG	2018	March – December 2017	Pakistan	Case-control study	Flow cytometry	40	15	4660 ± 4300	11424 ± 9042	Leptin
Afarid M	2018	February 2016 – February 2017	Iran	Case-control study	ELISA	44	39	40 ± 33	19 ± 14	Leptin
Katagiri M	2018	August 2012 – June 2013	Tokyo	Case-control study	ELISA	25	8	356 ± 419	47 ± 49	Leptin
Wu R	2020	December 1, 2018 – November 30, 2019	China	Case-control study	ELISA	140	75	7.83 ± 2.46	4.22 ± 1.04	Apelin
Yang HS	2021	March 2020 – February 2021	Korea	Case-control study	Flow cytometry	59	39	72373 ± 496855	7524 ± 7316	Leptin
Wang L	2021	–	China	Case-control study	ELISA	45	45	35 ± 10	22 ± 10	Chemerin
Yasir M	2022	August 2016 – March 2018	India	Case-control study	ELISA	112	56	1584 ± 1335.48, 451 ± 205.4, 3.35 ± 3.18	993 ± 955, 377 ± 180, 6.43 ± 4.29	Apelin, chemerin, omentin-1

### Results of the meta-analysis

**Figure 3:**
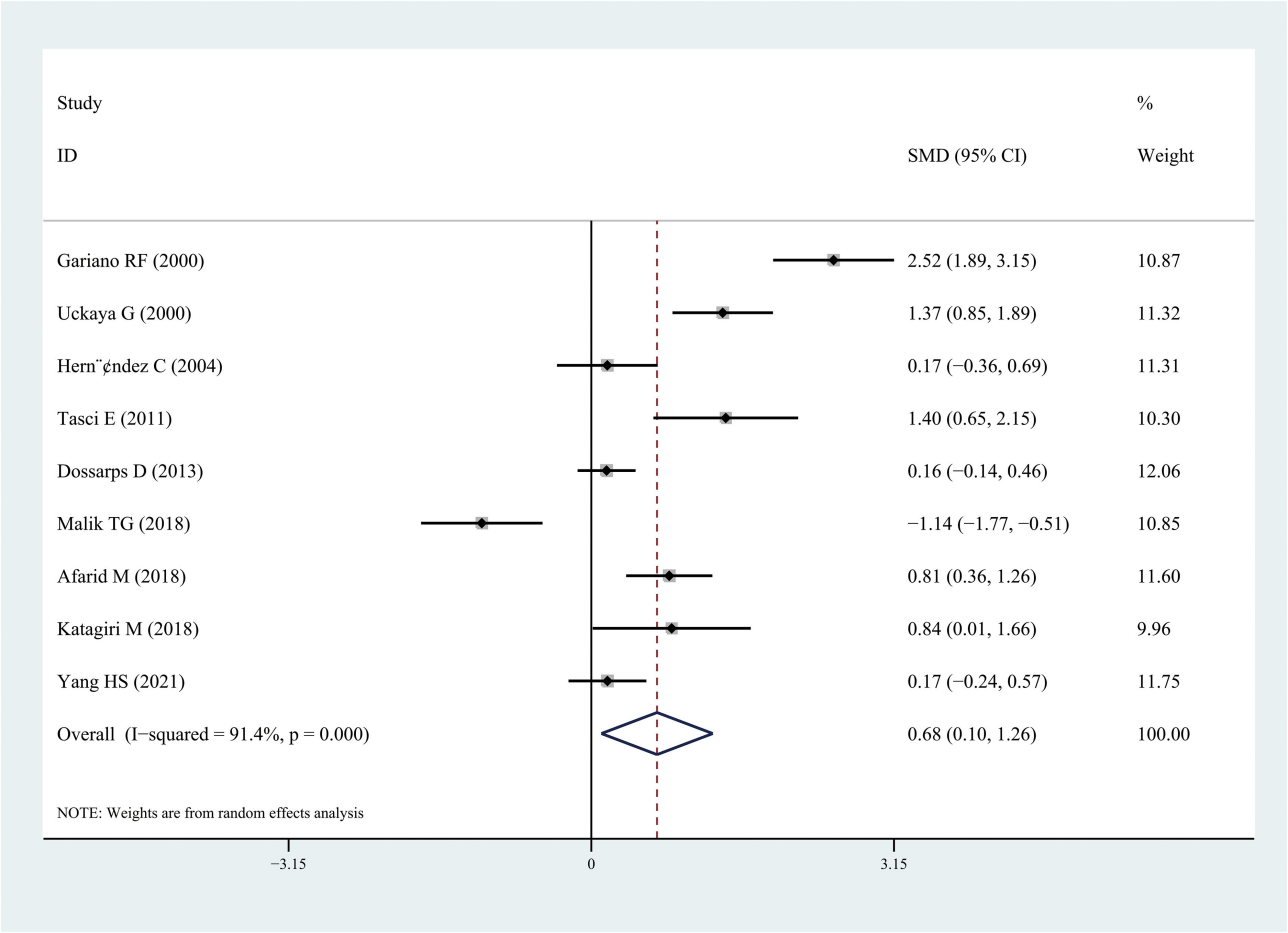
Forest plots of leptin level in patients with diabetic retinopathy compared with controls. Diamond represents the SMDs at 95% CI. SMD, standardized mean difference; CI, confidence interval.

**Figure 4:**
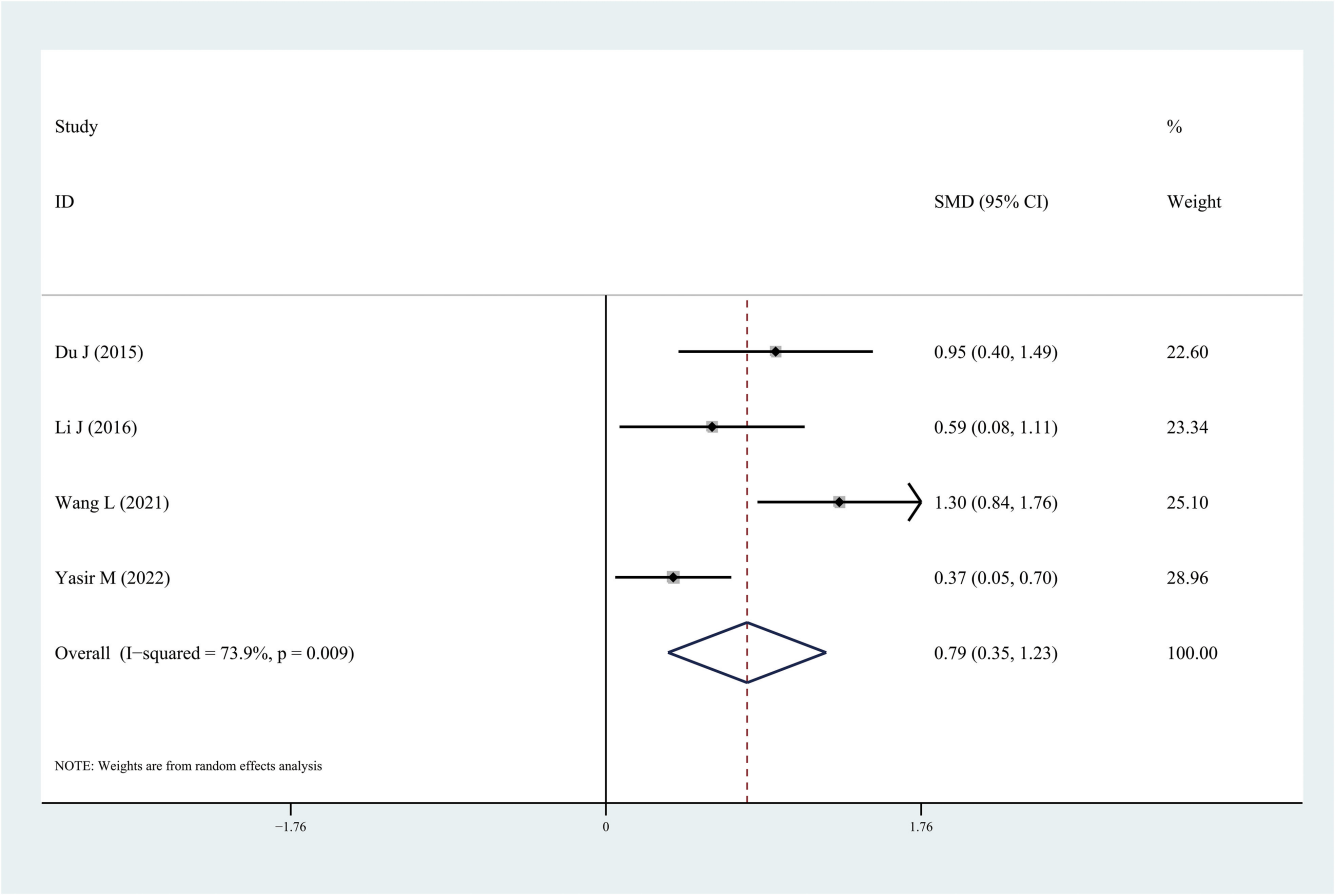
Forest plots of chemerin level in patients with diabetic retinopathy compared with controls. Diamond represents the pooled SMDs at 95% CI. SMD, standardized mean difference; CI, confidence interval.

**Figure 5:**
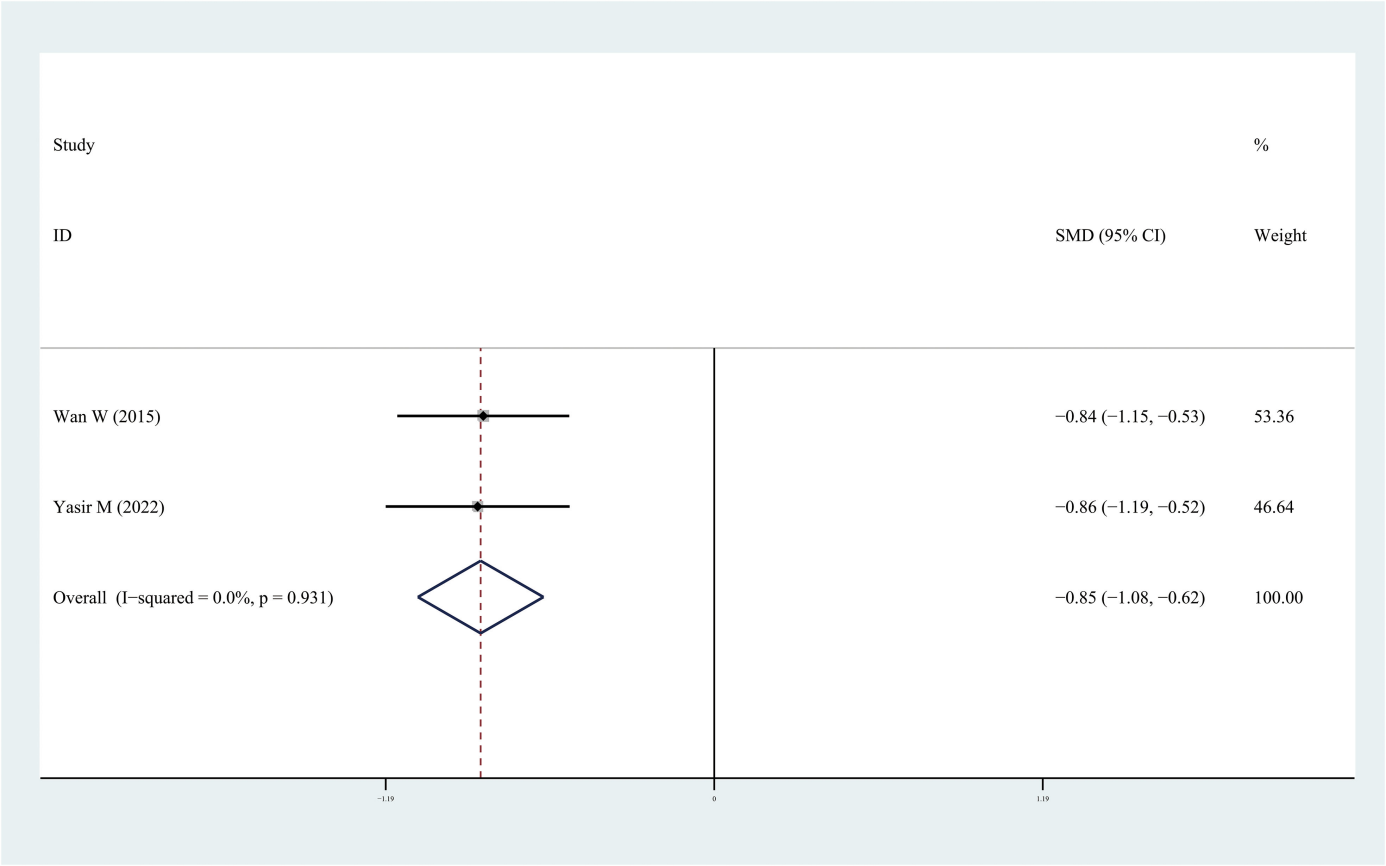
Forest plots of omentin-1level in patients with diabetic retinopathy compared with controls. Diamond represents the pooled SMDs at 95% CI. SMD, standardized mean difference; CI, confidence interval.

**Figure 6:**
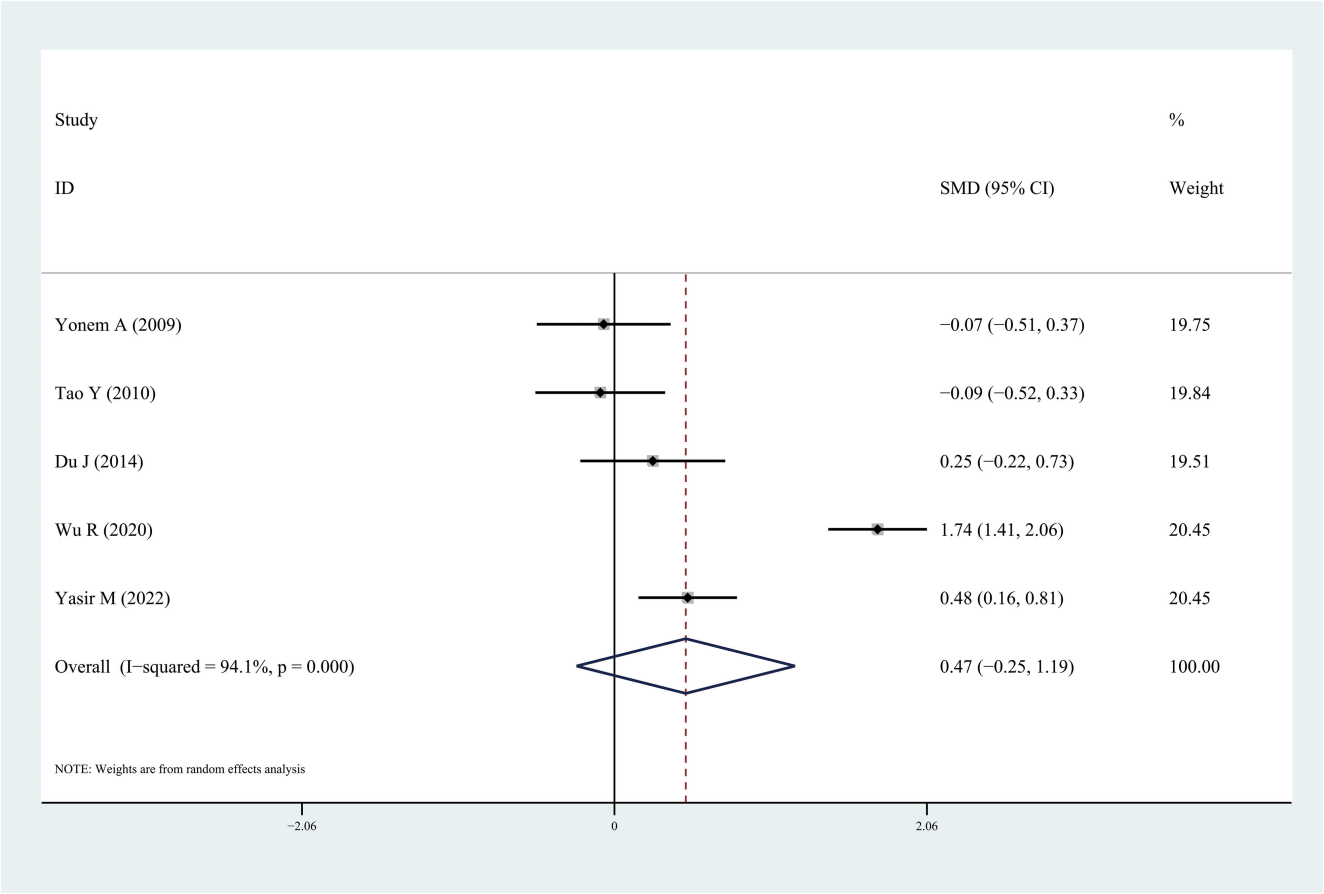
Forest plots of apelin level in patients with diabetic retinopathy compared with controls. Diamond represents the pooled SMDs at 95% CI. SMD, standardized mean difference; CI, confidence interval.

The results of the meta-analysis demonstrated that the leptin and chemerin levels in patients with DR were significantly higher than those in non-DR patients (SMD: 0.68, 95% CI [0.1, 1.26]; SMD: 0.79, 95% CI [0.35, 1.23]); the forest funnel plots are presented in **Figures 3**, **4**. The omentin-1 level in patients with DR was significantly lower than that in the controls (SMD: –0.85, 95% CI [–1.08, –0.62]) (**Figure 5**). However, the apelin levels in patients with DR were higher than those in the controls; however, no significant difference was observed (SMD:0.47, 95% CI [–0.25, 1.19]) (**Figure 6**). In addition, these associations were highly heterogeneous.

### Sensitivity analysis and publication bias

Using sensitivity analysis by excluding individual studies one by one, the results showed little difference, suggesting that the results of this study were relatively credible. A comprehensive search of the articles obtained from the databases was performed. Begg’s and Egger’s tests were also performed and the results revealed that the possibility of publication bias was small.

## Discussion

This meta-analysis is the first to evaluate the leptin, apelin, chemerin, and omentin-1 levels in patients with DR. Some studies have examined the levels of these adipocytokines in patients with DR; however, owing to inconsistent results, determining the relationship between these adipocytokines and DR is challenging. In this meta-analysis, 18 independent studies were included and analyzed. The results demonstrated that the leptin and chemerin levels in patients with DR were significantly higher than those in the controls (SMD: 0.68, 95% CI [0.1, 1.26]; SMD: 0.79, 95% CI [0.35, 1.23]). The omentin-1 levels in patients with DR were significantly lower than those in the controls (SMD: –0.85, 95% CI [–1.08, –0.62]). However, the apelin levels in patients with DR were higher than those in the controls; however, no significant difference was observed.

Leptin, which is a single-chain protein molecule containing 167 amino acid residues, is synthesized, and secreted by the adipose tissue. The main function of leptin is to regulating energy metabolism via the central nervous system. It regulates appetite through a negative feedback system and completes processes, such as reducing appetite, inhibiting energy intake, promoting energy consumption, and reducing fat synthesis, by activating receptors in target cells (10). Leptin can inhibit the secretion of insulin in patients with diabetes, thereby, affecting the insulin-mediated vascular endothelial dilation function (VEDF). Evidence suggests that the leptin levels in the vitreous body are associated with neovascular ophthalmopathies (11). Leptin can stimulate Signal transducer and activator of transcription 3 (STAT3) in the retinal endothelial cells, thereby increasing STAT3 phosphorylation and vascular endothelial growth factor (VEGF) mRNA expression in the vascular endothelium, which may be the molecular mechanism that induces retinal neovascularization (31). Leptin receptors are present in the fibrovascular tissue outside the retina of patients with diabetes. Kimura et al. demonstrated that leptin can increase the level of nitric oxide in the blood (32). Leptin induces endothelial cell proliferation by promoting endothelin secretion and mitosis. Park et al. suggested that leptin could regulate the reorganization of the vascular matrix by upregulating the expression of matrix metalloproteinases and promoting mitosis of vascular endothelial cells, thus participating in the formation of new blood vessels (33).

Chemerin, also known as retinoic acid receptor response protein-2, was first identified as a coding product of tazarotene-induced gene-2 in psoriatic lesions (34). In 2007, Bozaoglu et al. determined chemerin as a new adipose factor that is highly expressed in adipocytes and associated with obesity and metabolic syndrome (35). Increasing evidence suggests that chemerin is frequently involved in neovascularization. Patients with PDR had significantly higher serum chemerin levels, and a positive correlation exists between chemerin and VEGF. The chemerin and CMKLR1 systems are implicated in mediating cellular migration following inflammation. Accumulating evidence has revealed that chemerin, similar to VEGF, inhibits angiogenesis and the formation of new blood vessels in human endothelial cells (36).

In 2003, omentin-1 was first isolated and identified from the visceral omental adipose tissue by Yang et al., who demonstrated that treatment with recombinant omentin-1 can enhance insulin-stimulated glucose transport, indicating that omentin-1 can improve insulin sensitivity and resistance (37). Omentin-1, which is a newly discovered secreted protein with insulin sensitivity, is associated with obesity and obesity-related diseases, such as insulin resistance and diabetes. There are two highly homologous omentins: omentin-1 and omentin-2. Among them, omentin-1 is the main circulating form in human plasma. According to reports, the omentin-1 levels are decreased in individuals with type 2 diabetes mellitus, metabolic syndrome, and obesity. DR is associated with angiogenesis. Endothelial cell migration and angiogenesis induced by VEGF are significantly reduced by omentin-1 (38, 39). Omentin-1 may serve as an important protective factor against the development of DR by acting as an antiangiogenic mediator. One study found that omentin inhibited the inflammatory response induced by tumor necrosis factor (TNF) in human endothelial cells and smooth muscle cells of the vascular system. Inflammatory cytokines, such as interleukin-6 and C-reactive protein, are inversely associated with serum omentin-1 levels. Inflammation may reportedly contribute to DR and omentin-1 may play an inhibitory role in the inflammatory pathways in DR (40, 41).

Apelin is a G protein-coupled receptor, which was discovered in 1993. It is homologous to the angiotensin II type 1 receptor and is called angiotensin receptor-like protein J (APJ). The *APJ* gene is located on 11q12, and the APJ protein is composed of 380 amino acids (42). Apelin plays an important role in the regulation of T2DM. Apelin can promote glucose and lipid metabolism, increase insulin sensitivity, reduce blood sugar levels, improve diabetes, and reduce the occurrence of diabetes by regulating cardiovascular function and reducing food intake (43). In this study, although an increase in the apelin levels was observed in patients with DR, no statistically significant differences were found.

However, this study has certain limitations. Owing to the lack of large sample sizes, most studies included in this meta-analysis had small sample sizes. Furthermore, many isomers of adipocytokines, such as apelin-12 and apelin-36, exist; some studies did not indicate which isomer was used. Different detection methods of adipocytokines levels have been used in these studies. These factors may have affected the results. Therefore, the results obtained herein should be interpreted with caution as further research is needed.

## Conclusion

To the best of our knowledge, this meta-analysis is the first to evaluate the leptin, chemerin, apelin, and omentin-1 levels in patients with DR. Our findings suggest that leptin, chemerin, and omentin-1 levels are significantly altered in patients with DR. Further high-quality studies are warranted to confirm the association between adipocytokines and DR.

## Data availability statement

The original contributions presented in the study are included in the article/**Supplementary Material**. Further inquiries can be directed to the corresponding authors.

## Author contributions

YJ: investigation, software, writing – original draft. HF: writing – original draft, formal analysis, project administration. JX: writing – original draft, resources, visualization. YX: writing – original draft, data curation. XS: conceptualization, writing – original draft.

## Supplementary material

Supplementary File S1 Preferred reporting items for systematic review and meta-analyses (PRISMA) checklist.
